# A Lyme Neuroborreliosis Case Presenting With Ophthalmoplegia, Dysarthria, and Spastic Quadriparesis

**DOI:** 10.7759/cureus.100825

**Published:** 2026-01-05

**Authors:** Maria Sklirou, Mohammad Aboulwafaali, Anusha Karunasagar, Sharmilee Gnanapavan, Ganesh Arunachalam

**Affiliations:** 1 Integrative Medicine, Princess Alexandra Hospital, Harlow, GBR; 2 Neurology, Princess Alexandra Hospital, Harlow, GBR; 3 Microbiology, Princess Alexandra Hospital, Harlow, GBR; 4 Geriatrics, Princess Alexandra Hospital, Harlow, GBR

**Keywords:** csf lymphocytosis, guillain barre syndrome (gbs), intravenous immunoglobulins (ivig), lyme neuroborreliosis, spastic quadriparesis

## Abstract

Lyme neuroborreliosis (LNB) represents the neurological manifestation of Lyme disease and is the most common form of disseminated Lyme infection in Europe. Central nervous system involvement in LNB includes cranial neuritis, meningitis, radiculoneuritis, encephalitis, and encephalomyelitis with quadriparesis. We report a patient presenting with complex ophthalmoplegia, speech impairment, and spastic quadriparesis. The neurological weakness initially followed an ascending pattern suggestive of Guillain-Barré syndrome (GBS). Cerebrospinal fluid (CSF) analysis revealed lymphocytic pleocytosis, and serum testing showed positivity for immunoglobulin G (IgG) antibodies to Borrelia P39 and variable major protein-like sequence expressed (VlsE) antigens. IgG and immunoglobulin M (IgM) immunoblots were also positive. The patient was initially treated with intravenous immunoglobulin (IVIG) for presumed GBS and subsequently received antibiotics following CSF findings indicative of central nervous system infection, with subsequent neurological improvement. This case highlights the diagnostic challenges of LNB and underscores the importance of thorough evaluation of clinical history, neurological examination, and laboratory investigations to achieve diagnostic accuracy.

## Introduction

Lyme borreliosis (LB) is a multisystem infectious disease caused by the *Borrelia burgdorferi sensu lato *(*Bb*)complex, a spirochete transmitted to humans through the bite of infected ticks [1,2]. Owing to its substantial prevalence across Europe, LB represents the most common vector-borne infection in the region [1]. The hallmark clinical feature of early localised disease is the characteristic erythema migrans rash, which is observed in approximately 80-94% of cases [1]. Nevertheless, the clinical spectrum of LB is remarkably broad, encompassing a variety of cardiac, musculoskeletal, and neurological manifestations [2].

Lyme neuroborreliosis (LNB) involves disseminated infection with neurological symptoms and constitutes the most frequent manifestation of disseminated LB [1]. Epidemiological studies conducted throughout Europe indicate that LNB occurs in approximately 3% of all LB cases, underscoring its relative rarity [1]. Central nervous system (CNS) involvement in LNB can present with diverse clinical syndromes, including cranial neuritis, meningitis, and radiculoneuritis [2]. Rare neurological presentations, reported in approximately 2-4% of LNB cases, include encephalitis and encephalomyelitis associated with quadriparesis [3-5]. The heterogeneity of these presentations frequently renders diagnosis challenging and necessitates a high index of clinical suspicion. Herein, we describe a rare case of LNB presenting with complex ophthalmoplegia, dysarthria, and spastic quadriparesis.

## Case presentation

A 64-year-old woman presented to the emergency department with a one-day history of acute, bilateral, and symmetrical global lower limb weakness. In the lower limbs, there was symmetrical, proximal and distal weakness, with a Medical Research Council (MRC) power grade of 4/5. In the three days preceding admission, she had experienced cough and malaise, which were treated in the community as a presumed lower respiratory tract infection. Approximately one week prior to hospitalisation, she also reported an episode of diarrhoea. Her past medical history was significant for left mastectomy, adrenal adenoma, hypertension, depression, and a perineural cyst. Notably, she had recently travelled to Madeira, Portugal.

Clinical progress

**Figure 1:**
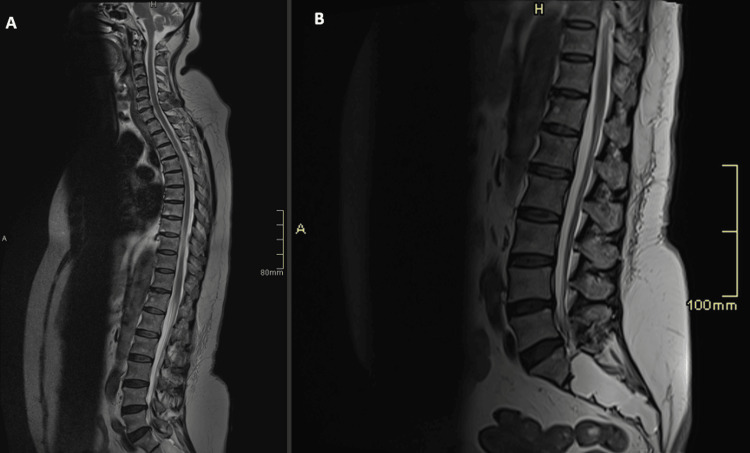
MRI whole spine and repeat MRI lumbar spine. Sagittal T2-weighted MR image of the whole spine, which was reported as a normal study (A). Sagittal T2-weighted MR image of the lumbar spine performed three weeks after the first study (A) demonstrated normal appearances and no discrete lesions. Incidentally, a large Tarlov cyst was seen in the sacral spinal canal, with no abnormal enhancement after administration of gadolinium (B).

**Figure 2:**
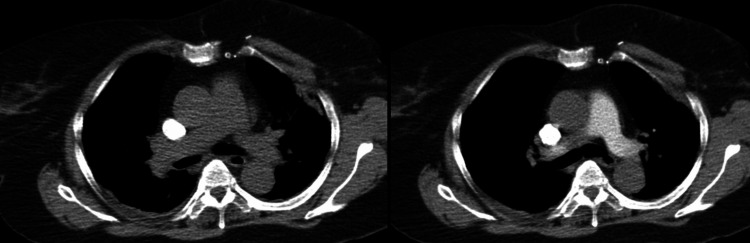
CT pulmonary angiogram. Multi-slice images from a CT pulmonary angiogram. The study was negative for pulmonary embolism.

The initial differential diagnosis included cauda equina syndrome and considered metastatic spinal cord compression, given her history of breast cancer. Magnetic resonance imaging (MRI) of the whole spine demonstrated no pathological findings consistent with either diagnosis (Figure 1). An elevated D-dimer level raised suspicion for pulmonary embolism (PE), which was subsequently excluded on computed tomography pulmonary angiography (CTPA) (Figure 2).

On the fourth day of admission, the patient's condition deteriorated. Patient developed confusion and dysarthria, accompanied by worsening neurological weakness following a profound ascending pattern that began to involve the hands. She also described paraesthesia--characterised as a “pins and needles” sensation--initially affecting the lower limbs in a non-dermatomal distribution and later extending to the hands. In addition, the patient reported new episodes of urinary incontinence. The patient became hypoxic, requiring up to four litres per minute of supplemental oxygen, and developed new-onset hypertension, with blood pressure reaching 210/105 mmHg.

Cranial nerve examination revealed bilateral ophthalmoparesis without pupillary involvement. No bulbar weakness was demonstrated. No other cranial nerve deficit was elicited on clinical examination. Neurological examination of the upper limbs demonstrated bilateral proximal weakness with a Medical Research Council (MRC) power grade of 4/5 and distal weakness graded at 3/5. This is compared with the normal power examination in the upper limbs at the time of admission. In the lower limbs, there was symmetrical, predominantly proximal weakness, mostly marked in the hip flexors, with an MRC power grade of 3/5. Weakness was noted in the distal muscle groups, with MRC power grade 4/5. This compares with symmetrical, global lower limb weakness both proximally and distally at the time of admission, with an MRC power grade of 4/5. The objective assessment of sensation was limited due to concomitant confusion. There was no discrete sensory level identified. Hyperreflexia was noted in the assessment of the upper and lower limbs' reflexes, and plantar responses were deemed equivocal. Evaluation of gait could not be performed due to the patient's inability to mobilise.

The constellation of rapidly ascending weakness, hypertensive emergency with features suggestive of hypertensive encephalopathy, and a recent diarrhoeal prodrome prompted urgent consideration of Guillain-Barré syndrome (GBS). Empirical treatment with intravenous immunoglobulin (IVIG) was initiated based on a presumed diagnosis of GBS with autonomic dysfunction and possible atypical features. Antibiotic therapy was concurrently commenced as per Trust guidelines to cover for potential bacterial and viral encephalitic pathogens after cerebrospinal fluid (CSF) analysis demonstrated lymphocytosis. Following a positive serum result for suspected Lyme borreliosis (LB), the patient was commenced on a three-week course of intravenous ceftriaxone, as per microbiology guidance.

A diagnosis of botulism was also considered, particularly given the antecedent diarrhoea, and discussed with the Gastrointestinal Bacteria Reference Unit (GBRU). However, the ascending pattern of paralysis was inconsistent with botulism and therefore excluded. Further consultation with the Rare and Imported Pathogens Laboratory (RIPL) indicated that CSF testing for LNB might yield inconclusive results following administration of IVIG.

Results

Serum analysis performed after the first dose of IVIG was positive for IgG antibodies against the Borrelia variable major protein-like sequence expressed (VlsE) antigen and immunoglobulin M (IgM) antibodies against the Borrelia P39 antigen (Table 1). Immunoblot testing for Borrelia IgM and immunoglobulin G (IgG) also returned positive results (Table 1). Lumbar puncture conducted after the second dose of IVIG revealed CSF lymphocytosis (Table 2). A repeat lumbar puncture performed six days later due to ongoing neurological weakness again demonstrated persistent CSF lymphocytosis (Table 2).

**Table 1:** Lumbar puncture results. The first lumbar puncture (LP) was performed after the third dose of intravenous immunoglobulin (IVIG). The second LP was performed after eight days of IVIG treatment and six days after the first LP. The CSF from the first LP demonstrates lymphocytosis. The culture result was negative in both LPs. Negative viral PCR. CSF: cerebrospinal fluid; PCR: polymerase chain reaction; HSV-1: Herpes simplex virus 1; HSV-2: Herpes simplex virus 2; VZV: Varicella zoster virus. Glucose fasting normal range: 3.0-7.7 mmol/l, CSF protein normal range: 0.15-0.45 g/l, CSF glucose normal range: 2.5-4.5 mmol/l.

Parameters evaluated	First LP	Second LP
Glucose non-fasting	8.9 mmol/l	13.4 mmol/l
CSF protein	1.28 mmol/l	0.97 mmol/l
CSF glucose	5.1 mmol/l	7.8 mmol/l
CSF WBC	108 Cmm	29 Cmm
CSF RBC	255 Cmm	48 Cmm
CSF polymorphs	8%	30%
CSF lymphocytes	92%	70%
Gram stain	No organisms seen	No organisms seen
Culture result	No growth after prolonged incubation	No growth after prolonged incubation
Parechovirus PCR	Not detected	Not detected
Enterovirus PCR	Not detected	Not detected
HSV1 & 2 DNA (PCR)	Not detected	Not detected
VZV DNA (PCR)	Not detected	Not detected
Adenovirus PCR	Not detected	Not detected
CSF appearance	Straw coloured	Clear and colourless

**Table 2:** Serological test results for Borrelia burgdorferi. Enzyme immunoassay results on the tested serum for *Borrelia burgdorferi*. The Borrelia IgG/IgM C6 EIA enzyme immunoassay was utilised. The serum tested positive for IgG antibody against the Borrelia V1sE antigen and IgM antibody against the Borrelia P39 antigen. The immunoblot for Borrelia IgM and IgG was also tested positive. The assay tested against following antigens in the serum: P83, P58, P43, P39, P30, OspC, p21, Osp17, DBPA, P14, V1sE, P41, P39, OspC, Osp17, V1sE. Bb: *Borrelia burgdorferi*, IgG: immunoglobulin G, IgM: immunoglobulin M, Ag: antigen, EIA: enzyme immunoassay, V1sE: variable major protein-like sequence expressed, DBPA: decorin-binding protein A.

Antibody/antigen test for Bb	Test result
Borrelia IgG/IgM C6 EIA	Positive
IgG Borrelia P83 Ag	Negative
IgG Borrelia P58 Ag	Negative
IgG Borrelia P43 Ag	Negative
IgG Borrelia P39 Ag	Negative
IgG Borrelia P30 Ag	Negative
IgG Borrelia OspC Ag	Negative
IgG Borrelia p21 Ag	Negative
IgG Borrelia Osp17Ag	Negative
IgG Borrelia DBPA Ag	Negative
IgG Borrelia P14 Ag	Negative
IgG Borrelia V1sE Ag	Positive
IgM Borrelia P41 Ag	Negative
IgM Borrelia P39 Ag	Positive
IgM Borrelia OspC Ag	Negative
IgM Borrelia Osp17Ag	Negative
IgM Borrelia V1sE Ag	Negative
IgG Borrelia DBPA Ag	Negative
Immunoblot for Borrelia IgM and IgG	Positive

**Figure 3:**
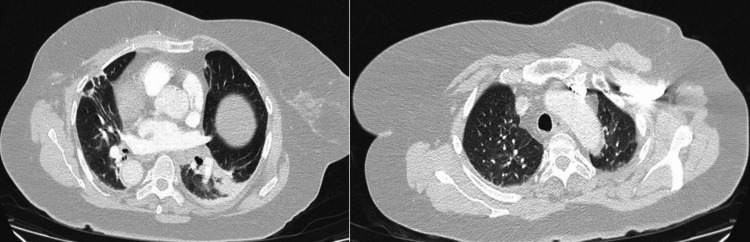
CT pulmonary angiogram with lung windows. Multi-slice images from a CT pulmonary angiogram with lung windows. In the lung windows, foci of consolidation were seen in the left upper lobe and bilateral lower lobes, most severely in the posterior segment of the right lower lobe. CT: computed tomography.

**Figure 4:**
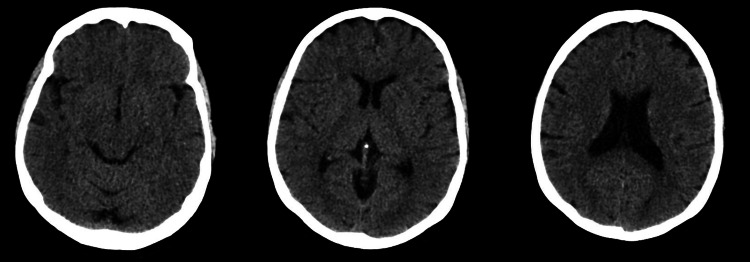
CT head. Multi-slice unenhanced CT images of the head. Normal study, with no evidence of acute intracerebral haemorrhage, major territorial infarct, space-occupying lesion, or hydrocephalus. CT: computed tomography.

**Figure 5:**
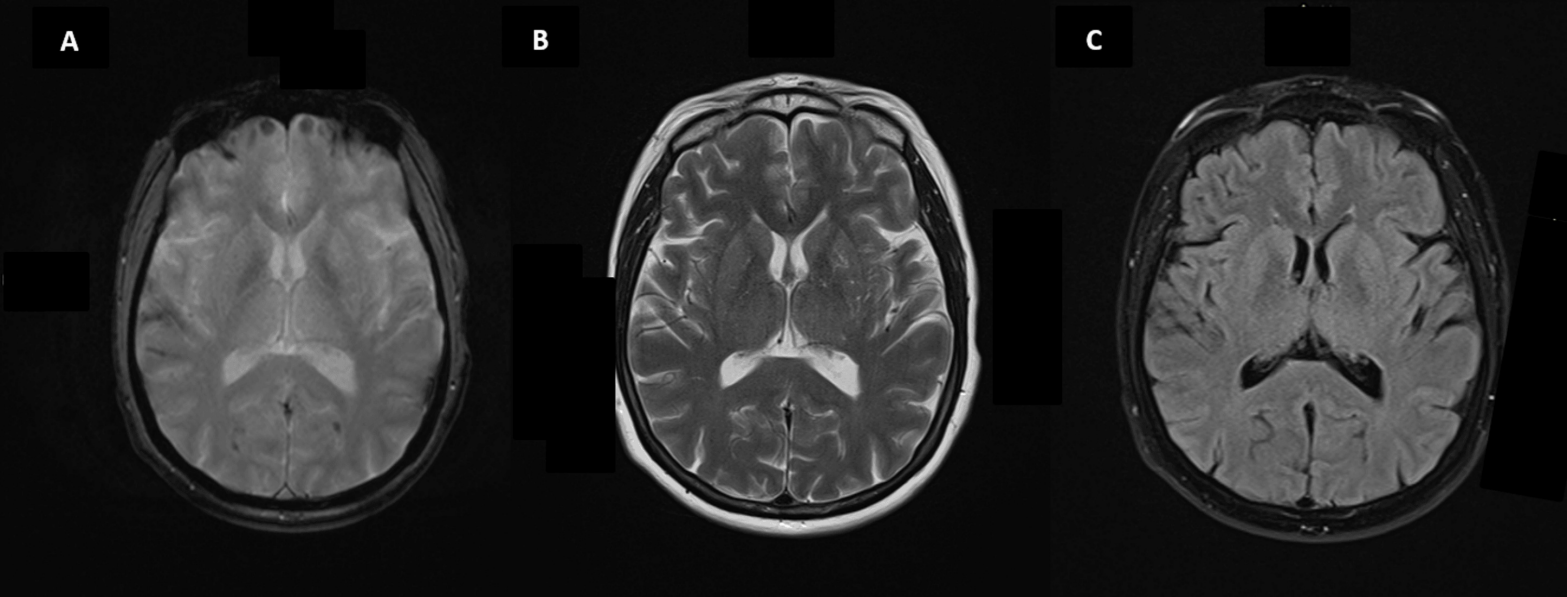
MRI head. Axial MR images of the brain, with gradient echo (A), T2-weighted (B), and T2 FLAIR (C) sequences. No evidence of intracranial metastasis, demyelination, or encephalitis was identified, but cortical haemosiderin staining was noted in the right temporal lobe and bilateral parieto-occipital sulci/cortex. In the absence of an acute subarachnoid bleed on the previous CT head study, this appearance raised the possibility of underlying cerebral amyloid angiopathy. FLAIR: fluid-attenuated inversion recovery; CT: computed tomography.

**Figure 6:**
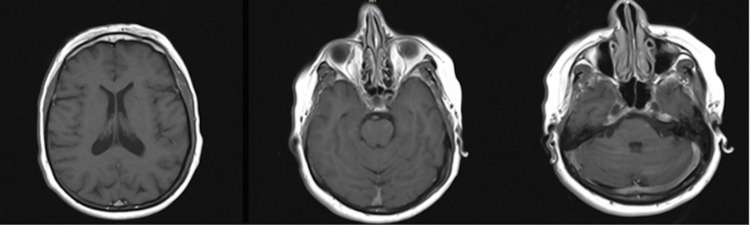
MRI head with contrast. Axial MR images of the brain, with contrast sequences. No evidence of intracranial metastasis, demyelination, or encephalitis was identified. No facial cranial nerve or other cranial nerve pathology was suspected.

Inflammatory markers showed normal white blood cell and neutrophil counts, with a mildly elevated C-reactive protein (CRP) of 20 mg/dL (reference <5 mg/dL). A respiratory viral panel from nasal and throat swabs was negative for infectious respiratory pathogens. Neuronal antibody screening--including anti-glutamic acid decarboxylase (GAD), acetylcholine receptor, and anti-muscle-specific tyrosine kinase (MUSC) antibodies--was negative. MRI of the entire spine revealed no vertebral metastasis or cauda equina compression, and a subsequent contrast-enhanced MRI of the lumbar spine showed no pathological abnormalities (Figure 1). CTPA excluded pulmonary embolism but revealed foci of consolidation in the left upper and both lower lobes, reported as possibly infective in nature (Figures 2, 3). Computed tomography (CT) of the brain on the day of admission demonstrated no acute intracranial abnormality (Figure 4). MRI of the brain, performed the following day after neurological deterioration, showed no evidence of intracranial metastasis, demyelination, or encephalitis (Figures 5, 6). Discussion of the MRI head images with the neuroradiologist confirmed the absence of any identifiable facial nerve or other cranial nerve deficits (Figure 6).

Patient outcomes

This case describes a suspected LNB presentation initially treated with IVIG due to a preliminary diagnosis of GBS. The patient showed neurological improvement following IVIG and antibiotic therapy during their inpatient stay. Patient progressed from quadriparesis to ambulation. At discharge, the patient was able to walk, occasionally using a frame when climbing stairs. The patient's speech disturbance had also resolved.

## Discussion

The diagnostic evaluation of central nervous system disorders is inherently intricate, necessitating a multiparametric strategy that synthesizes a comprehensive clinical history--including pertinent travel exposures--with defining clinical features and corroborative laboratory investigations [2]. In this case, the initial differential diagnosis encompassed infectious, inflammatory, and paraneoplastic aetiologies. Cervical myelopathy was considered at an early stage, and metastatic spinal cord compression was specifically interrogated; however, both were excluded through neuroimaging, with MRI of the entire spine reported as normal (Figure 1). The differential diagnosis of spastic quadriparesis is extensive, incorporating intracranial pathologies such as stroke, neoplasm, and demyelinating disease, all of which were excluded on neuroimaging. Infective encephalitic processes were entertained as potential aetiological substrates for the patient's spastic quadriparesis, prompting initiation of empirical antimicrobial therapy. In view of the progressive neurological decline and the pronounced ascending trajectory of weakness, GBS remained a principal consideration, and the patient was empirically managed with IVIG. Subsequent CSF analysis demonstrated pleocytosis, a finding congruent with an infectious process and regarded as atypical for GBS.

The diagnosis of LNB, in this case, is supported by a combination of patient history, clinical findings, and laboratory evidence. First, the presence of spastic quadriparesis represents a recognised feature of late-stage LNB. Second, alternative differential diagnoses were systematically excluded; GBS was considered improbable due to preserved reflexes and CSF pleocytosis, which is atypical for GBS. The CSF analysis showed pleocytosis, which is consistent with established diagnostic criteria for LNB [6]. MRI of the brain revealed no encephalitic or neoplastic changes, and spinal imaging demonstrated no evidence of compressive myelopathy. Third, immunoassay results confirmed positivity for both IgG and IgM antibodies against LNB antigens, including IgG directed against the VlsE antigen and IgM against the P39 antigen, further supporting the diagnosis of neuroborreliosis. This was corroborated by positive immunoblot findings for both IgM and IgG. These results are consistent with prior studies demonstrating that late immune responses in Lyme disease are mediated by IgG against the VlsE antigen, detectable in more than 90% of positive sera [7]. Collectively, these findings substantiate exposure to *Borrelia burgdorferi*, the pathogen responsible for the clinical syndrome described. Based on the combination of clinical findings and investigative results, LNB was deemed the most probable diagnosis. This case represents a complex manifestation of neuroborreliosis, presenting with ophthalmoplegia, spastic quadriparesis following an ascending pyramidal pattern of weakness, and speech disturbance. Such variability reflects the wide clinical spectrum of LNB and its diagnostic challenges. Importantly, the absence of a recalled tick bite or cutaneous manifestations does not preclude an LB diagnosis [2,6,7]. Literature indicates that only a small percentage, between 40-50% of patients infected with Lyme disease, are able to recall a tick bite [6,8]. Similarly, a minority of patients are able to recall skin manifestations [6]. In this instance, the patient reported insect bites during her recent travel, initially attributed to mosquito exposure.

The pathological manifestations of LNB are broadly divided into early and late disease stages, involving neurological processes that affect both the CNS and the peripheral nervous system (PNS) [6]. Clinical features of both stages may include encephalitis, encephalomyelitis, and, notably, quadriparesis, while gait disturbance is a well-recognised sequela in late LNB [6]. This aligns with the present case, which illustrates a presentation of LNB with spastic quadriparesis. Late LNB is exceedingly rare, representing approximately 5% of all LB cases [6]. Encephalitis is recognised as a late feature of LNB and remains an uncommon manifestation [9]; data from a Scandinavian cohort study reported its prevalence as low as 3.3% [10]. Furthermore, speech and language disorders have been described within the encephalitic spectrum [7]. Our case featured quadriparesis and dysarthria, consistent with this spectrum. A previously published report also documented an acute LNB presentation with transient aphasia [11]. In the present case report, the patient demonstrated findings consistent with bilateral ophthalmoparesis. Neuro-ophthalmological manifestations have likewise been reported in association with LNB [12]. Frequent ophthalmological manifestations encountered in literature comprised of palpebral diastasis secondary to facial palsy, blurred vision, double vision, and sixth nerve impairment [12]. This case report illustrates ophthalmoparesis as a rare clinical presentation within the spectrum of neuroborreliosis. The patient developed new episodes of urinary incontinence by day four of admission, a feature that may indicate an evolving myelopathic process. Nonetheless, the initial MRI of the whole spine and the repeat MRI of the lumbar spine performed three weeks after presentation demonstrated no evidence of myelitis, with post-contrast sequences showing no abnormal enhancement (Figure 1). Myelopathic presentations of Lyme neuroborreliosis are rare, with approximately 7% of cases exhibiting myelitis [13]. The presence of inflammatory CSF changes, demonstrated by pleocytosis, further supports LNB-associated myelitis. Collectively, these observations underscore the exceptional nature of the clinical presentation, marked by ophthalmoparesis, dysarthria, quadriparesis, and urinary incontinence, and emphasise the necessity of including LNB in the differential diagnosis of complex neurological syndromes.

The capacity of LNB to mimic GBS has been well documented [14-16]. The combination of ascending neurological weakness and paraesthesia following a diarrhoeal prodrome initially suggested GBS, consistent with a prior report describing Lyme disease as a potential GBS mimic [16]. In addition, due to the findings consistent with ophthalmoplegia, a diagnosis of Miller Fisher Syndrome (MFS) was also considered, which is a GBS variant. However, several atypical features argued against a diagnosis of GBS, including the preservation of reflexes, the rapid onset of weakness reaching its nadir within 24 hours, and CSF findings of pleocytosis being inconsistent with classic GBS [17]. Given the potentially severe consequences of untreated neuroborreliosis with profound CNS involvement, this case reinforces the importance of considering Lyme disease in patients presenting with GBS-like features.

Limitations

The definitive diagnosis of LNB remains a well-recognised clinical challenge [18]. According to the European Federation of Neurological Societies (EFNS), three criteria are recommended for establishing a diagnosis of LNB: (i) neurological symptoms consistent with LNB in the absence of an alternative explanation, (ii) CSF pleocytosis, and (iii) intrathecal production of *Borrelia burgdorferi *antibodies [6]. Fulfillment of all three criteria indicates a definitive diagnosis, whereas the presence of two supports a probable diagnosis. In contrast, American diagnostic criteria do not require intrathecal evidence of Borrelia antibodies for a definitive diagnosis [6]. In the present case, the third EFNS criterion was not met. Consultation with the RIPL indicated that further CSF testing for LNB might be inconclusive following IVIG administration; therefore, additional testing was not pursued. The lack of international consensus on diagnostic criteria, compounded by the diverse clinical manifestations of LNB, continues to pose significant diagnostic difficulties [6]. Moreover, intrathecal antibody detection has been reported to have a sensitivity as low as 55% [6].

It is acknowledged that the serum sample yielding positive *Borrelia burgdorferi* antibody results was obtained after administration of the first IVIG dose. Seroconversion following IVIG therapy has been described in the literature [19]. However, antibody detection secondary to IVIG does not necessarily correlate with the development of clinical disease. Notably, Hanson et al. [20] reported in a cohort study examining serological profiles before and after IVIG administration that cumulative IVIG doses exceeding 100 g were associated with new serological findings. In this case, the positive serum result for Lyme borreliosis was obtained before administration of the second IVIG dose, when the cumulative dose was 34.8 g, which is well below the reported threshold for likely assay interference. Additionally, as IVIG preparations consist primarily of pooled IgG with only trace amounts of IgM [20], the detection of both IgM and IgG antibodies in this case further supports a true immunological response to *Borrelia burgdorferi*.

## Conclusions

This case report illustrates a complex presentation of LNB manifesting with intricate ophthalmoplegia and spastic quadriparesis. It also demonstrates symptom improvement following the administration of intravenous immunoglobulin (IVIG) and antibiotics. The case resembled GBS, with an ascending pattern of pyramidal weakness; however, due to the presence of hyperreflexia, GBS was considered unlikely. IVIG was administered in response to the rapid progression of weakness and presumed autonomic dysfunction occurring alongside a hypertensive emergency.

The patient made a clinical recovery following treatment with IVIG and ceftriaxone for three weeks, in accordance with trust protocol. Neurological function improved from quadriparesis at admission to resumption of baseline walking ability by the time from hospital discharge. This presentation underscores the diagnostic challenges associated with LNB and highlights the importance of thorough evaluation of clinical history, physical signs, and investigative findings to achieve an accurate diagnosis. Furthermore, this case supports existing evidence that Lyme disease can mimic GBS and reinforces the need to consider LNB in the differential diagnosis of complex neurological presentations. 
